# Visual Field Progression in Childhood Glaucoma Versus Open-Angle Glaucoma: A Retrospective Comparative Study

**DOI:** 10.3390/jcm15031146

**Published:** 2026-02-02

**Authors:** Ainhoa Colina-Jareno, Ruben Sanchez-Jean, Irene Serrano-Garcia, Julian Garcia-Feijoo, Carmen Mendez-Hernandez

**Affiliations:** 1 Ophthalmology Department, Hospital Clinico San Carlos, Institute of Health Research (IdISSC), 28040 Madrid, Spain; ainhoa.colina@salud.madrid.org (A.C.-J.); rsjean@salud.madrid.org (R.S.-J.); jgarciafeijoo@hotmail.com (J.G.-F.); 2 Unit of Methodological Research Support, Hospital Clinico San Carlos, Institute of Health Research (IdISSC), 28040 Madrid, Spain; isgarcia@salud.madrid.org; 3 Department of Immunology, Ophthalmology and ORL, Ramon Castroviejo Institute for Ophthalmic Research, Complutense University of Madrid, 28040 Madrid, Spain

**Keywords:** childhood glaucoma, open-angle glaucoma, visual field progression, survival analysis, mean defect, regression analysis, long-term outcomes

## Abstract

**Background:** Evidence on long-term visual field progression in childhood glaucoma compared with open-angle glaucoma (OAG) is limited. We compared the rate and timing of visual field progression and identified predictors of final visual field status. **Methods:** Single-center, retrospective, observational study including childhood glaucoma and OAG, with ≥3 reliable visual field tests and ≥2 years of follow-up. Visual fields were obtained with Octopus perimeter (Haag-Streit Diagnostics, Köniz, Switzerland) with the G grid and TOP strategy. Visual field progression was evaluated using the rate of change in mean defect (MD, dB/year). Rates were compared with the Mann–Whitney U test. Timing was evaluated with Kaplan–Meier and restricted mean survival time (RMST). Cox models assessed risk of progression. Secondary analysis used multiple linear regression to identify predictors of final MD. The mean follow-up duration was 5.7 ± 2.6 years. **Results:** 171 eyes (87 childhood glaucoma, 84 OAG) were analyzed. Childhood glaucoma had worse baseline MD (10.7 ± 7.5 dB) than OAG (5.1 ± 6.5 dB, *p* < 0.001), and underwent more surgeries, while OAG used more medications. The median MD progression rate was −2.3 dB/year [IQR: −5.6 to 0.1] in childhood glaucoma vs. 0.0 dB/year [IQR: −1.2 to 1.3] in OAG (*p* < 0.001), a value consistent with functional stability under treatment, with some eyes showing negative slopes indicating relative improvement. In Octopus perimetry, MD is expressed on a positive scale, so a negative slope reflects absence of visual field worsening, suggesting comparatively greater deterioration in OAG. Kaplan–Meier curves showed similar progression-free survival between groups (Log-Rank *p* = 0.284). RMST at 12 years was 10.93 years in childhood glaucoma and 10.56 years in OAG (difference ≈ 4.4 months, not clinically relevant). These survival results should be interpreted cautiously due to the low number of progression events and the high censoring rate. In regression, baseline MD was the strongest predictor of final MD; a higher number of medications was associated with worse final MD; number of surgeries and follow-up duration were not significant predictors. **Conclusions:** MD slopes suggested faster deterioration in OAG than in childhood glaucoma, whereas the timing to first progression was similar between groups. Baseline differences and treatment patterns were consistent with functional stability in childhood glaucoma under current management strategies. These findings support individualized follow-up and timely intervention, especially in pediatric patients.

## 1. Introduction

Visual field perimetric assessment is one of the most important evaluations in glaucoma patients for determining both disease diagnosis and progression [1,2]. The current gold standard in perimetry is Standard Automated Perimetry, also known as White-on-White perimetry. Computerized automated perimeters provide short test durations, which are suitable for patients who may easily lose concentration. This increases the reproducibility and reliability of the test, making the results more dependable [3,4,5,6]. The implementation of artificial intelligence in perimetric software may assist physicians in the early detection of glaucomatous damage or disease progression [7].

Detecting visual field progression is essential for understanding glaucoma, assessing disease stability, and determining the degree of deterioration considered clinically significant for each patient [8]. A minimum of five reliable visual fields is required to detect visual field progression. The rate of progression usually requires at least two years of follow-up. Previous studies recommend that newly diagnosed patients should undergo at least three reliable visual field tests after diagnosis [9,10,11,12].

There is limited available literature specifically addressing visual field progression in childhood glaucoma; pediatric perimetry presents limitations in terms of reliability and frequency of testing, and therefore many studies and expert consensus reports prioritize the control of intraocular pressure (IOP) and the structural evaluation of both the optic nerve and the nerve fiber layer thickness (RNFL) using optical coherence tomography (OCT), and consider the Mean Defect (MD) to be the most relevant functional index when analyzing visual field data [13,14,15,16,17].

The purpose of this study is to compare the rate and timing of visual field progression between patients with childhood glaucoma and those with open-angle glaucoma (OAG), and to describe the clinical and treatment characteristics of both groups during long-term follow-up.

The secondary objective is to assess the association between antiglaucoma treatment at the end of follow-up—number of glaucoma surgeries and antiglaucomatous medications—and the final visual field status, adjusting for baseline MD, diagnosis (childhood glaucoma vs. OAG), and follow-up duration.

## 2. Material and Methods

### 2.1. Study Design and Population

This was a single-center, retrospective, observational study conducted at Hospital Clínico San Carlos, Madrid, in accordance with the Declaration of Helsinki and approved by the institutional Ethics Committee (protocol number 20/287-E). Patients were eligible if they had a confirmed diagnosis of childhood glaucoma (including primary congenital and juvenile glaucoma) or OAG, with a minimum follow-up of two years and at least three reliable visual field tests after diagnosis. Only patients with reliable visual fields, as determined by the examiner, were included. For each patient, one visual field per year was selected for analysis. Exclusion criteria were unreliable visual fields (high rates of false positives, false negatives, or fixation losses), incomplete clinical data, follow-up shorter than two years, or absence of at least three reliable visual field tests.

### 2.2. Data Collection

The following variables were collected from medical records for each patient: age, gender, central corneal thickness (pachymetry), pseudophakia status, number and type of glaucoma surgeries, number and type of antiglaucomatous medications, best-corrected visual acuity (BCVA) at the last follow-up visit, initial and final IOP measured using applanation tonometry with a Perkins tonometer (Haag-Streit AG, Köniz, Switzerland). IOP values were not adjusted for central corneal thickness. All patients included in the study had an established diagnosis of glaucoma prior to the study period, and the IOP measurements reported correspond to treated follow-up values rather than diagnostic pressures. Initial and final retinal nerve fiber layer thickness (RNFL) measured by Spectral-domain optical coherence tomography (SD-OCT) using the Heidelberg Spectralis system (Heidelberg Engineering, Heidelberg, Germany). OCT examinations were obtained systematically during the same periodic follow-up visits in which visual fields were performed, as part of the routine clinical protocol of our Glaucoma Unit. Visual field MD, OCT-derived RNFL measurements, and all ophthalmic examination data were recorded during the follow-up visits, in accordance with the routine clinical protocol of our Glaucoma Unit.

### 2.3. Visual Field Assessment and Progression Criteria

Visual field testing was performed using an Octopus perimeter (Haag-Streit Diagnostics, Köniz, Switzerland) with the G grid and TOP strategy. Octopus perimetry using G program and TOP strategy was selected because it is routinely used in our Glaucoma Unit and ensures homogeneity of longitudinal visual field data. The TOP algorithm provides rapid threshold estimation with significantly shorter test duration, higher patient tolerability—especially important in pediatric examinations—and robust reproducibility of results, making it particularly suitable for long-term follow-up in both childhood and adult glaucoma patients.

One reliable visual field per year was included for analysis in all patients, and this criterion was applied equally to both childhood glaucoma and OAG groups, ensuring comparable follow-up frequency between groups.

In Octopus perimetry, the MD is expressed on a positive scale, where more positive values indicate greater visual field loss; therefore, a negative MD progression slope reflects relative functional improvement rather than deterioration. Accordingly, a positive MD slope indicates functional worsening.

To minimize the learning effect, the first two visual fields of each patient were excluded from the analysis. Accordingly, the baseline visual field used for analysis corresponded to the third reliable test. Visual field progression was defined as a worsening of ≥0.6 dB in MD between the initial and final visual field tests. The rate of progression (dB/year) was calculated for each eye as the difference between final and initial MD divided by the years of follow-up. In this context, a positive MD progression rate would indicate a deterioration of the visual field, whereas a negative MD progression would reflect a relative functional improvement.

### 2.4. Statistical Analysis

Both eyes from each patient were included as units of analysis when eligible. Although this approach is common in retrospective ophthalmic studies, it introduces within-subject correlation that has not been accounted for in the analyses. Confidence intervals and *p*-values may be optimistic due to the lack of adjustment for inter-eye correlation. Descriptive statistics were used for demographic and clinical variables, presented as mean ± standard deviation (SD), median [interquartile range, IQR], or frequency (percentage), as appropriate. Comparisons of baseline continuous variables between groups were performed using the independent samples t-test or the Mann–Whitney U test, depending on the distribution assessed by the Shapiro–Wilk test for normality. Categorical variables were compared using the chi-square test or Fisher’s exact test, as appropriate.

The rate of visual field progression (dB/year) was compared between groups using the Mann–Whitney U test. Boxplots were used to illustrate the distribution of progression rates. Survival analysis was performed using the Kaplan–Meier method to compare progression rates between groups, with the log-rank (chi-square) test used to assess the equality of survival distributions. The restricted mean survival time (RMST) was calculated as the area under the Kaplan–Meier survival curves up to 10 and 12 years, providing an interpretable measure of time free from progression when the median was not reached.

Cox proportional hazards regression models were used to evaluate the association between glaucoma diagnosis (OAG vs. childhood glaucoma) and the risk of visual field progression. The primary model included diagnosis and baseline IOP as covariates, both measured at study entry. Age was not included as a covariate in the main model, as it is inherently linked to the diagnostic category and its inclusion could introduce overadjustment bias. Additional models were constructed as sensitivity analyses, incorporating age or baseline MD as alternative covariates. The proportional hazards assumption was assessed for all models.

Analysis included only cases with complete data, and the number of cases in each model is specified in the results. Covariates were selected based on clinical relevance and significant differences between groups in baseline characteristics.

As a secondary objective, we assessed the association between antiglaucomatous treatment at the end of follow-up and the final visual field MD. A multiple linear regression analysis was performed with the final MD as the dependent variable. Independent variables included baseline MD, diagnosis (childhood glaucoma vs. primary open-angle glaucoma), follow-up duration (years), number of antiglaucomatous medications, and number of glaucoma surgeries. All predictors were entered simultaneously using the ENTER method. Both eyes from each patient were included as units of analysis. Model outputs reported coefficients (B), standard errors, standardized coefficients (Beta), t-statistics, two-sided *p*-values, and 95% confidence intervals. Residual normality and collinearity were assessed using residual plots and variance inflation factors.

All statistical tests were two-sided, and a *p*-value < 0.05 was considered statistically significant. All analyses were conducted using SPSS version 30.0 (IBM Corp., Armonk, NY, USA).

## 3. Results

Data from 171 eyes of 86 patients were analyzed: 87 eyes of 44 patients with childhood glaucoma and 84 eyes of 42 patients with OAG.

The mean age of patients with childhood glaucoma was 22.9 ± 10.0 years (range, 10–46), whereas adult patients with OAG were significantly older, with a mean age of 71.1 ± 11.5 years (range, 47–90; *p* < 0.001). Women represented 50.0% of patients with OAG and 40.2% of those with childhood glaucoma, with no statistically significant difference between groups (*p* = 0.129).

At baseline, patients with childhood glaucoma showed a higher MD (10.7 ± 7.5 dB vs. 5.1 ± 6.5 dB, *p* < 0.001) compared with patients with OAG. RNFL thickness (74.5 ± 22.2 µm vs. 76.9 ± 22.0 µm, *p* = 0.279), central corneal thickness (556.0 ± 55.1 µm vs. 542.7 ± 36.9 µm, *p* = 0.096), and IOP (18.2 ± 4.6 mmHg vs. 18.7 ± 4.7 mmHg, *p* = 0.240) were comparable between both groups.

Pseudophakia was more frequent in OAG (50.0%) than in childhood glaucoma (8.0%; *p* < 0.001). Patients with childhood glaucoma were receiving a lower number of topical hypotensive medications (1.1 ± 1.3 vs. 1.4 ± 1.0, *p* = 0.044) and had undergone a significantly greater number of antiglaucoma surgical procedures (1.8 ± 1.6 vs. 0.6 ± 0.7, *p* < 0.001). Glaucoma-related surgical procedures were performed in 71.3% of childhood glaucoma patients and 52.9% of OAG patients. Topical antiglaucoma medications, including prostaglandin analogues, beta-blockers, carbonic anhydrase inhibitors, and α-agonists were more frequent in adults (74.1%) than in children (48.3%). Final BCVA was lower in the childhood glaucoma group, although the difference was not statistically significant. The childhood glaucoma group had a higher proportion of patients with severe visual impairment (counting fingers, amaurosis, or light perception) at the end of follow-up.

The median MD progression rate was −2.3 dB/year [IQR: −5.6 to 0.1] in childhood glaucoma and 0.0 dB/year [IQR: −1.2 to 1.3] in OAG (*p* < 0.001), noting that the negative MD progression in childhood glaucoma reflects relative functional improvement in Octopus perimetry. The median rate of 0.0 dB/year in the OAG group reflects overall functional stability under treatment. The interquartile range (−1.2 to 1.3 dB/year) corresponds to expected test–retest variability in perimetry, and small negative slopes in some eyes represent relative functional improvement due to variability. The median RNFL thickness change was 0.0 [−1.0 to 1.0] µm in childhood glaucoma and 0.0 [−1.7 to 0.0] µm in OAG (*p* < 0.001). The mean duration of follow-up was similar between groups (5.9 ± 2.5 years in childhood glaucoma vs. 5.5 ± 2.6 years in OAG, *p* = 0.388).

Table 1 summarizes the baseline demographic and clinical characteristics of both groups.

**Figure 1:**
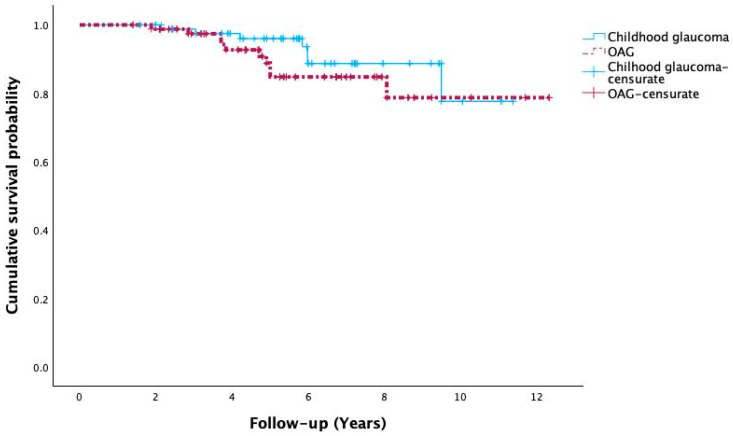
Kaplan–Meier survival curves for visual field progression in childhood glaucoma and open-angle glaucoma (OAG). The figure shows the cumulative probability of remaining free from visual field progression over up to 12 years of follow-up. The blue line represents childhood glaucoma, and the red dashed line represents OAG. Censored cases are indicated by plus signs. Both groups exhibited similar progression-free survival, with no statistically significant difference between curves (log-rank *p* = 0.284).

Kaplan–Meier survival curves for visual field progression are shown in Figure 1.

A total of 17 events occurred, and 154 cases were censored (90.1%). In the childhood glaucoma group, there were 87 eyes with 7 events and 80 censored cases (92.0%), while in the OAG group, there were 84 eyes with 10 events and 74 censored cases (88.1%).

Table 2 summarizes Kaplan–Meier survival estimates for childhood glaucoma and OAG.

The probability of remaining free from progression at 5 years was: childhood glaucoma 0.960 (95% CI 0.915 to 1.000) and OAG 0.848 (95% CI 0.754 to 0.942); at 10 years: childhood glaucoma 0.776 (95% CI 0.559 to 0.993) and OAG 0.787 (95% CI 0.644 to 0.930).

The Log-Rank test showed no significant differences between curves (χ^2^ = 1.147; df = 1; *p* = 0.284). The median time free from progression was not reached in either group. Restricted mean survival times (RMST) were 10.44 years (95% CI 9.79 to 11.09) in childhood glaucoma and 10.82 years (95% CI 9.94 to 11.69) in OAG. Overall, these data suggest a low progression rate under this threshold and no statistically significant differences between groups during the evaluated period.

The interpretation of these findings is limited by the very small number of progression events (*n* = 17) and the high censoring rate (>90%), conditions that substantially reduce statistical power and may account for the absence of statistically significant differences between groups. RMST estimates are presented as descriptive measures without inferential interpretation.

The results of the Cox proportional hazards models are presented in Table 3.

In the primary model, which included glaucoma diagnosis (OAG vs. childhood glaucoma) and baseline IOP as covariates, neither variable was significantly associated with the risk of visual field progression. The hazard ratio (HR) for OAG compared to childhood glaucoma was 1.83 (95% CI: 0.69 to 4.84; *p* = 0.221), and the HR for baseline IOP (per mmHg) was 1.06 (95% CI: 0.96 to 1.17; *p* = 0.263).

Sensitivity analyses were performed using alternative models that included age or baseline MD as covariates. In the model with age, the HR for OAG was 0.81 (95% CI: 0.09 to 7.35; *p* = 0.853), and for age (per year) was 1.02 (95% CI: 0.97 to 1.06; *p* = 0.458). In the model with baseline MD, the HR for OAG was 1.69 (95% CI: 0.60 to 4.74; *p* = 0.321), and for baseline MD (per dB) was 1.00 (95% CI: 0.93 to 1.07; *p* = 0.941). None of the models showed statistically significant associations between the covariates and the risk of progression. These results are shown in Table A1 of Appendix A.

Table 4 shows the estimation of the restricted mean survival time (RMST) up to 12 years for both groups.

The RMST was 10.93 years in childhood glaucoma and 10.56 years in OAG. Child glaucoma group spent on average 4.4 months free from progression within the first 12 years. Survival probabilities at 12 years were S (12) = 0.776 for childhood glaucoma and 0.787 for OAG.

### MD Progression Rate (dB/year)

The distribution of MD progression rates differed significantly between groups. Median (IQR) rates were −2.3 dB/year (−5.6 to 0.1) in childhood glaucoma and 0.0 dB/year (−1.25 to 1.27) in OAG, indicating faster worsening in OAG and, on average, stability or improvement in childhood glaucoma. The Mann–Whitney U test confirmed a statistically significant difference (*p* < 0.001). In about 7 out of 10 cases, an eye with OAG is more likely to worsen faster than an eye with childhood glaucoma. These results indicate that visual field deterioration tends to occur more frequently and more rapidly in OAG than in childhood glaucoma.

To address the secondary objective, a multiple linear regression analysis was performed. In the multivariable linear regression model including baseline MD, diagnosis (OAG vs. childhood glaucoma), follow-up duration, number of antiglaucomatous medications, and number of glaucoma surgeries, the model was significant (*p* < 0.001) and explained 56.5% of the variance in final MD (R^2^ = 0.565; adjusted R^2^ = 0.551; SEE = 5.13 dB). Baseline MD was the strongest predictor of final MD (B = 0.770, 95% CI 0.656 to 0.885; *p* < 0.001). The number of medications showed a negative association with final MD (B = −0.770, 95% CI −1.459 to −0.080; *p* = 0.029). Diagnosis was positively associated with final MD (B = 2.699, 95% CI 0.903 to 4.496; *p* = 0.003), consistent with higher final MD in adult glaucoma relative to childhood glaucoma when adjusting for covariates. The number of surgeries (B = 0.323, 95% CI −0.302 to 0.948; *p* = 0.309) and follow-up duration (B = −0.051, 95% CI −0.361 to 0.258; *p* = 0.745) were not significantly associated with final MD. The results of the regression model are presented in Table 5.

## 4. Discussion

This study provides a comprehensive comparison of visual field progression between childhood glaucoma and OAG in adults, with long-term follow-up. Despite a worse baseline visual MD and a higher number of surgeries during follow-up in the childhood glaucoma group, the rate of visual field progression over time was similar to that observed in adults with OAG. These findings are consistent with previous reports suggesting that, with appropriate management, children with glaucoma can achieve functional stability comparable to adults, even though their initial damage is often more severe [1,2,3,16,17,18,19,20,21,22]. It is important to highlight that the Octopus TOP G1 perimeter expresses the MD in positive values. A more negative MD slope reflects relative improvement rather than deterioration, in contrast to Humphrey perimetry. In this context, the childhood glaucoma group, which showed a median progression rate of −2.3 dB/year, experienced relative functional improvement during follow-up. In contrast, the OAG group, with a median progression rate of 0.0 dB/year, did not show such improvement, resulting in comparatively worse functional status at the end of follow-up.

The apparent improvement in MD values observed in the childhood glaucoma group, from 10.69 to 7.60 dB, could be related to visual field variability and improved reliability over time, particularly in younger patients. Although the first two visual fields were excluded to minimize learning effects, some residual improvement in test performance and treatment-related stabilization might also contribute to better MD values during follow-up. The median MD slope of 0.0 dB/year observed in the OAG group indicates functional stability rather than progression. The fact that the interquartile range included both slightly negative and slightly positive values is consistent with the normal variability of longitudinal perimetry. Modest negative slopes should therefore be interpreted as fluctuations related to test–retest variability or improved reliability over time.

The higher frequency of surgical intervention in the pediatric group, as observed in our study, aligns with the literature, where surgery is often the mainstay of treatment for childhood glaucoma, while adults are more frequently managed with medical therapy [7,16,17,23]. Despite more frequent surgeries, the functional outcomes in children were stable, and the rate of progression did not differ significantly from adults, supporting the effectiveness of early and aggressive surgical management [16,17,24].

Visual field testing in children presents unique challenges, including the learning effect and variability in test reliability [8,22]. Our methodology, which excluded the first two visual fields, is supported by previous studies to minimize this effect [4,22]. The use of MD as the primary outcome is also consistent with current recommendations for both pediatric and adult glaucoma progression studies [4,8].

The progression rates observed in our study are in line with those reported in large cohorts and meta-analyses of glaucoma patients, where the majority of treated patients show slow progression, but a minority experience rapid or catastrophic loss [5,13,20]. In our series, the cumulative probability of progression at 10 years was approximately 22% in childhood glaucoma and 21% in adults, which is comparable to the rates reported in other long-term studies [13,16,22].

The secondary objective of this study was to identify predictors of final visual field status. Our multiple linear regression confirmed that baseline MD was the strongest predictor of final MD, in line with prior evidence showing that baseline visual field involvement and early MD trends anticipate subsequent global deterioration [18,19,20]. A higher number of topical medications was associated with worse final MD, likely reflecting greater disease severity or treatment complexity. Worse adherence has been linked to greater MD loss over time, and multi-drop treatments are associated with poorer compliance [21,22]. Diagnosis also influenced outcomes. Juvenile and childhood glaucoma often requires early surgical management and differs from adult OAG in IOP behavior and medication responsiveness, supporting adjusted differences in final functional status between groups [17]. Neither the number of surgeries nor follow-up duration independently predicted final MD after adjustment; functional decline after surgery appears more related to IOP fluctuation, and time alone does not determine final MD once baseline severity and IOP control are considered [23,24,25].

Risk factors for visual field progression in glaucoma include higher baseline IOP, greater initial visual field damage, older age, and certain ocular comorbidities [11,12,14]. In childhood glaucoma, additional factors such as the presence of nystagmus, media opacities, and anterior segment dysgenesis have been identified as predictors of poor outcomes [7,16]. Our findings confirm that, although children start with worse visual function, their progression can be controlled with timely intervention.

Structural assessment with OCT, particularly RNFL thickness, is increasingly used to complement functional testing in glaucoma progression [1,2,3,4,9,10]. In our study, RNFL thickness remained stable in both groups, supporting the notion that structural and functional stability can be achieved with appropriate management [2]. OCT provides objective, reproducible measurements of structural damage, with low variability when performed by trained technicians. Recent developments in artificial-intelligence–assisted OCT analysis have further improved segmentation precision, noise filtering, and the detection of subtle structural changes. These advances are expected to further enhance reliability in the coming years. In contrast, visual field testing remains inherently subjective, depending on patient cooperation, attention, and fatigue. For this reason, structural OCT data remain a valuable complement to visual fields, particularly in pediatric patients and in cases where functional variability may be more pronounced.

Our results also highlight the importance of individualized follow-up strategies. Several studies emphasize that the frequency and duration of visual field testing are critical for detecting progression, especially in pediatric patients where cooperation and test reliability may vary over time [7,8,10,15]. Recent advances in artificial intelligence and machine learning are beginning to aid in the early detection of rapid progressors, which may further improve outcomes in both pediatric and adult populations [9,14].

Finally, our findings reinforce the need for a multidisciplinary approach in the management of childhood glaucoma, integrating surgical, medical, and rehabilitative strategies to optimize visual outcomes and quality of life [7,16,17,25]. The ongoing development of new surgical techniques and pharmacological agents, as well as improvements in diagnostic technologies, hold promise for further reducing the burden of visual impairment in this vulnerable population [16,17,25].

The discrepancy between progression rate and time to progression is consistent with the high censoring rate and the small number of events, 17, which reduce the power of the Log-Rank test and widen confidence intervals at later time points. Using the predefined progression threshold (≥0.6 dB worsening of MD between tests), childhood glaucoma eyes showed a slower rate of deterioration, but similar progression-free times compared to OAG. It is possible that applying a stricter criterion for progression—such as considering a slope of 0.1–0.3 dB/year—might have yielded different results. Future studies should explore this approach. Our cutoff was selected in accordance with the progression rate used in the Early Manifest Glaucoma Trial (EMGT) [26].

When we explored the risk of progression using Cox regression models, no significant association was found between diagnosis and time to progression. This result is consistent with the Kaplan–Meier curves, which showed similar survival patterns and were influenced by the high proportion of censored cases and the small number of events. Age was not included in the main Cox model because it strongly overlaps with diagnosis and could distort the estimates. Sensitivity analyses confirmed that, with so few progression events, the results remain unstable. For this reason, we relied on restricted mean survival time to describe long-term outcomes when the median was not reached, and these differences should be interpreted as descriptive.

This study has some important limitations. Its retrospective design, the small number of progression events combined with a high censoring rate, and the inclusion of both eyes from each patient in all analyses may affect the robustness of the findings. Confidence intervals and *p*-values may be overly optimistic due to the lack of adjustment for within-subject correlation. For this reason, the associations described should be interpreted with caution. Future studies should consider statistical methods that account for the correlation between eyes such as mixed-effects models or cluster-robust standard errors or restrict the analysis to one eye per patient.

Overall, our findings provide additional insight into long-term visual field outcomes in childhood glaucoma and suggest that individualized management strategies, including timely surgical intervention when appropriate, may play an important role in preserving visual function in pediatric patients.

## 5. Conclusions

MD slopes suggested faster deterioration in OAG than in childhood glaucoma, whereas the timing to first progression was similar between groups. Baseline MD strongly predicted final outcomes; more medications were linked to worse final MD, whereas surgeries and follow-up duration were not significant predictors. Overall, these results are consistent with the functional stability observed in childhood glaucoma under current management strategies. These findings support individualized long-term monitoring and timely intervention, particularly in pediatric glaucoma. Future prospective studies should account for intra-patient correlation.

## Figures and Tables

**Table 1:** Baseline Demographic and Clinical Characteristics. Values are presented as mean ± SD, median [IQR], or *n* (%), as appropriate.

Variable	Childhood Glaucoma	OAG (Adult Glaucoma)	*p*-Value
Age (years) *	22.92 ± 10.04	71.07 ± 11.49	<0.001
Female, *n* (%)	35/87 (40.2)	42/84 (50.0)	0.129
CCT (µm) *	555.96 ± 55.15	542.68 ± 36.90	0.096
Pseudophakia, *n* (%)	7/87 (8.04)	42/84 (50.0)	<0.001
Last BCVA *	0.68 ± 0.30	0.74 ± 0.23	0.083
First visit IOP (mmHg) *	18.18 ± 4.59	18.65 ± 4.68	0.240
Last visit IOP (mmHg) *	16.14 ± 3.68	16.55 ± 3.81	0.258
Initial RNFL (µm) *	74.54 ± 22.21	76.94 ± 21.99	0.279
Final RNFL (µm) *	74.45 ± 22.16	74.37 ± 21.35	0.491
RNFL change (µm) **	0.0 [−1.0 to 1.0]	0.0 [−1.75 to 0.0]	<0.001
Initial MD (dB) *	10.69 ± 7.46	5.40 ± 6.69	<0.001
Final MD (dB) *	7.60 ± 8.38	5.40 ± 6.70	0.029
MD slope (dB/year)**	−2.3 [−5.6 to 0.1]	0.0 [−1.25 to 1.27]	<0.001
No. of glaucoma surgeries *	1.77 ± 1.64	0.64 ± 0.67	<0.001
No. of glaucoma meds *	1.08 ± 1.26	1.38 ± 1.03	0.090
Years of follow-up *	5.88 ± 2.47	5.54 ± 2.64	0.388

Abbreviations: CCT, central corneal thickness; BCVA, best-corrected visual acuity; IOP, intraocular pressure; RNFL, retinal nerve fiber layer thickness; MD, mean defect; No, number; Meds, medications; (*), values are mean ± SD; (**), values are median [IQR]; SD, standard deviation; IQR, interquartile range.

**Table 2:** Kaplan–Meier Survival Estimates. Survival probability at 5 and 10 years for childhood glaucoma and OAG, based on annual MD progression ≥ 0.6 dB/year. Values include standard error (SE) and 95% confidence intervals (CI).

Time (Years)	Group	SP (S(t))	SE	95% CI Lower	95% CI Upper
5	Childhood glaucoma	0.960	0.023	0.915	1.000
5	OAG	0.848	0.048	0.754	0.942
10	Childhood glaucoma	0.776	0.111	0.559	0.993
10	OAG	0.787	0.073	0.644	0.930

OAG, open angle glaucoma; SP, S(t), survival probability indicates the probability of remaining free from visual field progression at each time point; SE, standard error; CI, confidence interval.

**Table 3:** Cox Proportional Hazards Model (Primary). Covariates: diagnosis (OAG vs. childhood glaucoma) and baseline intraocular pressure (IOP). Values are hazard ratios (HR), 95% confidence intervals (CI), and *p*-values.

Variable	HR (Exp (B))	95% CI Lower	95% CI Upper	*p*-Value
Diagnosis (OAG vs. CG)	1.834	0.694	4.841	0.221
Baseline IOP (per mmHg)	1.060	0.958	1.172	0.263

N = 171; Events = 17.

**Table 4:** Restricted Mean Survival Time (RMST) at 12 years. RMST (area under the Kaplan–Meier survival curve) estimated up to 12 years (τ = 12) for childhood glaucoma (CG) and primary open-angle glaucoma (OAG). Values shown as years; S (12) denotes the survival probability at 12 years.

Group	RMST at 12 Years (Years)	S (12)
Childhood glaucoma (CG)	10.93	0.776
Primary open-angle glaucoma (OAG)	10.56	0.787

Difference CG − OAG in RMST (12): 0.365 years (≈4.4 months). Note: RMST was computed by summing the stepwise areas under the Kaplan–Meier curve up to τ = 12 years. Results as descriptive; no confidence intervals or hypothesis tests for RMST differences.

**Table 5:** Multiple linear regression of final visual field mean defect (MD). Dependent variable: final MD (dB). Predictors entered simultaneously (ENTER). Values are unstandardized coefficients (B), standard errors (SE), standardized coefficients (Beta), t-statistics, two-sided *p*-values, and 95% confidence intervals (CI).

Predictor	B (Unstandardized)	SE	Beta (Standardized)	t	*p*-Value	95% CI (Lower)	95% CI (Upper)
Constant	−0.070	1.425	—	−0.049	0.961	−2.883	2.743
Baseline MD	0.770	0.058	0.758	13.334	<0.001	0.656	0.885
Years of follow-up	−0.051	0.157	−0.017	−0.326	0.745	−0.361	0.258
No. of medications	−0.770	0.349	−0.116	−2.204	0.029	−1.459	−0.080
No. of surgeries	0.323	0.316	0.058	1.020	0.309	−0.302	0.948
Diagnosis†	2.699	0.910	0.177	2.967	0.003	0.903	4.496

Model summary: R = 0.751; R^2^ = 0.565; adjusted R^2^ = 0.551; standard error of estimate (SEE) = 5.130 dB; ANOVA F (5, 164) = 42.550, *p* < 0.001. Abbreviations: MD, mean defect; SE, standard error; CI, confidence interval; SEE, standard error of estimate. †Diagnosis (OAG vs. childhood glaucoma).

## Data Availability

Data are available from the corresponding author on reasonable request and subject to institutional approvals.

## Appendix A

**Table A1:** Sensitivity Analyses (Cox Models).

Model S1: Covariates = Diagnosis (OAG vs. CG) and Age (Years)				
Variable	HR (Exp (B))	95% CI Lower	95% CI Upper	*p*-Value
Diagnosis (OAG vs. CG)	0.812	0.090	7.352	0.853
Age (per year)	1.017	0.973	1.064	0.458
**Model S2: Covariates = diagnosis (OAG vs. CG) and baseline MD (per dB)**				
**Variable**	**HR (Exp (B))**	**95% CI Lower**	**95% CI Upper**	*p* **-value**
Diagnosis (OAG vs. CG)	1.687	0.601	4.738	0.321
Baseline MD (per dB)	0.997	0.931	1.069	0.941

## References

[1] Fitzke F.W., Hitchings R.A., Poinoosawmy D., McNaught A.I., Crabb D.P. (1996). Analysis of Visual Field Progression in Glaucoma. Br. J. Ophthalmol..

[2] Wu Z., Medeiros F.A. (2018). Recent Developments in Visual Field Testing for Glaucoma. Curr. Opin. Ophthalmol..

[3] Tan J.C.K., Kalloniatis M., Phu J. (2023). Frontloading SITA-Faster Can Increase Frequency and Reliability of Visual Field Testing at Minimal Time Cost. Ophthalmol. Glaucoma.

[4] Tan J.C.K., Agar A., Kalloniatis M., Phu J. (2024). Quantification and Predictors of Visual Field Variability in Healthy, Glaucoma Suspect, and Glaucomatous Eyes Using SITA-Faster. Ophthalmology.

[5] Morales J., Weitzman M.L., González de la Rosa M. (2000). Comparison between Tendency-Oriented Perimetry (TOP) and Octopus Threshold Perimetry. Ophthalmology.

[6] Rowe F., Wishart M., Spencer S. (2014). Perimetry Comparisons for Octopus G Top and Dynamic Programmes versus Humphrey 24-2 SITA Fast and SITA Standard Programmes. Ophthalmol. Res. Int. J..

[7] Lan C.-H., Chiu T.-H., Yen W.-T., Lu D.-W. (2025). Artificial Intelligence in Glaucoma: Advances in Diagnosis, Progression Forecasting, and Surgical Outcome Prediction. Int. J. Mol. Sci..

[8] Chauhan B.C., Garway-Heath D.F., Goñi F.J., Rossetti L., Bengtsson B., Viswanathan A.C., Heijl A. (2008). Practical Recommendations for Measuring Rates of Visual Field Change in Glaucoma. Br. J. Ophthalmol..

[9] Crabb D.P., Russell R.A., Malik R., Anand N., Baker H., Boodhna T., Bronze C., Fung S.S., Garway-Heath D.F., Glen F.C. (2014). Frequency of Visual Field Testing When Monitoring Patients Newly Diagnosed with Glaucoma: Mixed Methods and Modelling. Health Serv. Deliv. Res..

[10] Ayala M. (2024). Risk Factors and Frequency of Examinations for Detecting Visual Field Deterioration in Patients with Newly Diagnosed Exfoliation Glaucoma in Sweden. J. Glaucoma.

[11] Keltner J.L., Johnson C.A., Levine R.A., Fan J., Cello K.E., Kass M.A., Gordon M.O. (2005). Normal Visual Field Test Results Following Glaucomatous Visual Field End Points in the Ocular Hypertension Treatment Study. Arch. Ophthalmol..

[12] Hu R., Racette L., Chen K.S., Johnson C.A. (2020). Functional Assessment of Glaucoma: Uncovering Progression. Surv. Ophthalmol..

[13] Patel D.E., Cumberland P.M., Walters B.C., Abbott J., Brookes J., Edmunds B., Khaw P.T., Lloyd I.C., Papadopoulos M., Sung V. (2022). Study of Optimal Perimetric Testing In Children (OPTIC): Developing Consensus and Setting Research Priorities for Perimetry in the Management of Children with Glaucoma. Eye.

[14] Naik A., Sihota R., Mahalingam K., Angmo D., Dada T., Kumar A., Kumar A., Gupta A. (2022). Evaluation of Visual Field Changes with Retinal Nerve Fiber Layer Thickness in Primary Congenital Glaucoma. Indian J. Ophthalmol..

[15] Hekmatjah N., Kumar A., Yu Y., Younessi D.N., Han Y., Ying G.-S., Oatts J.T. (2024). Visual Field Testing Frequency and Associations in Children with Glaucoma. J. Glaucoma.

[16] Brynskov T., Bach-Holm D., Kappelgaard P., Siersma V., Pedersen K.B., Kessel L. (2024). Long-term Functional and Structural Outcomes in Patients with Primary Congenital Glaucoma—A Danish Nationwide Study. Acta Ophthalmol..

[17] Weinreb R.N. (2013). Childhood Glaucoma.

[18] Garg A., De Moraes C.G., Cioffi G.A., Girkin C.A., Medeiros F.A., Weinreb R.N., Zangwill L.M., Liebmann J.M. (2018). Baseline 24-2 Central Visual Field Damage Is Predictive of Global Progressive Field Loss. Am. J. Ophthalmol..

[19] Medeiros F.A., Jammal A.A. (2023). Validation of Rates of Mean Deviation Change as Clinically Relevant End Points for Glaucoma Progression. Ophthalmology.

[20] Forchheimer I., de Moraes C.G., Teng C.C., Folgar F., Tello C., Ritch R., Liebmann J.M. (2011). Baseline Mean Deviation and Rates of Visual Field Change in Treated Glaucoma Patients. Eye.

[21] Newman-Casey P.A., Niziol L.M., Gillespie B.W., Janz N.K., Lichter P.R., Musch D.C. (2020). The Association between Medication Adherence and Visual Field Progression in the Collaborative Initial Glaucoma Treatment Study. Ophthalmology.

[22] Cvenkel B., Kolko M. (2022). Devices and Treatments to Address Low Adherence in Glaucoma Patients: A Narrative Review. J. Clin. Med..

[23] Khaliliyeh D., De Gainza A., Morales E., Caprioli J. (2023). Long-Term Visual Field Outcomes After Ahmed Glaucoma Valve Implantation. Am. J. Ophthalmol..

[24] Rao A., D’Cruz R. (2023). Visual Field Progression After Glaucoma Surgery in Pseudoexfoliation versus Primary Glaucoma. Clin. Ophthalmol..

[25] Shin Y.I., Jeong Y., Huh M.G., Kim Y.K., Park K.H., Jeoung J.W. (2024). Longitudinal Evaluation of Advanced Glaucoma: Ten Year Follow-up Cohort Study. Sci. Rep..

[26] Heijl A., Leske M.C., Hyman L., Bengtsson B., Hussein M. (2002). Reduction of Intraocular Pressure and Glaucoma Progression. Arch. Ophthalmol..

